# Automated segmentation and classification of lumbar transverse ultrasound views using a two-stage deep learning method

**DOI:** 10.3389/fradi.2026.1721194

**Published:** 2026-02-12

**Authors:** Jiangang Chen, Qing Yuan, Haowen Wang, Qiming Huang, Xueli Wang, Yiyan Xu, Dongpo Wei, Xiuyu Zheng, Caibao Hu, Dandan Zhang, Xulei Cui

**Affiliations:** 1 Shanghai Key Laboratory of Multidimensional Information Processing, School of Communication & Electronic Engineering, East China Normal University, Shanghai, China; 2 Department of Anesthesiology, Peking Union Medical College Hospital, Chinese Academy of Medical Sciences and Peking Union Medical College, Beijing, China; 3 College of Robotics Science and Engineering, Taiyuan University of Technology, Taiyuan, China; 4 School of Advanced Computing and Artificial Intelligence, Xi’an Jiaotong-liverpool University (Taicang Campus), Taicang, China; 5 Clinical Research Center for Precision Medicine of Abdominal Tumor of Fujian Province, Xiamen, China; 6 Xiamen Hospital Information Department of Zhongshan Hospital Affiliated to Fudan University, Shanghai, China; 7 School of Advanced Technology, Xi’an Jiaotong-liverpool University, Suzhou, China; 8 Department of Critical Care Medicine, Shanghai General Hospital, Shanghai Jiao Tong University School of Medicine, Shanghai, China; 9 Shenzhen Institute for Drug Control, Shenzhen Testing Center of Medical Devices, Shenzhen, Guangdong, China; 10 Department of Critical Care Medicine, Zhejiang Hospital, Hangzhou, Zhejiang, China

**Keywords:** classification, mid-lumbar ultrasound views, paramedian transverse foraminal view, paramedian transverse view, ultrasound image segmentation

## Abstract

**Background:**

Ultrasound guidance is widely used in lumbar regional anesthesia and chronic pain management because it provides radiation-free, portable, and real-time visualization. Among lumbar ultrasound views, the paramedian transverse view at the transverse process (PTV-TP) and the paramedian transverse foraminal view (PTFV) are essential for procedures such as lumbar plexus blocks and transforaminal injections. However, reliable recognition of these views remains technically challenging, particularly for novice clinicians.

**Objective:**

This study aimed to develop and evaluate a deep learning–based two-stage framework for automated detection and classification of PTV-TP and PTFV in lumbar ultrasound images.

**Methods:**

Ultrasound images were collected from 425 patients undergoing pain treatment at Peking Union Medical College Hospital. The proposed framework consisted of two stages. First, a U-Net–based semantic segmentation model was used to identify key lumbar anatomical landmarks, namely the transverse process and intervertebral foramen, which were grouped into a single class to reduce annotation complexity and improve robustness. Second, coordinate-based spatial features were extracted from the segmentation masks and classified using a support vector machine (SVM) to distinguish between PTV-TP and PTFV views. An end-to-end CNN-based classifier trained under the same experimental protocol served as a comparison. Segmentation performance was evaluated using the intersection-over-union (IoU) metric, and classification performance was assessed using accuracy.

**Results:**

The proposed U-Net–based segmentation achieved an IoU of 83.21%, outperforming conventional multi-class U-Net segmentation (77.33%). Based on the segmentation results, the coordinate-based SVM classifier achieved an accuracy of 97.66% in differentiating PTV-TP and PTFV views, which was substantially higher than that of the end-to-end CNN classifier (85.00%). Visualization results demonstrated stable performance across representative ultrasound images, including challenging imaging conditions.

**Conclusion:**

The proposed two-stage framework, which integrates semantic segmentation with coordinate-based classification, enables accurate automated recognition of lumbar ultrasound views. By explicitly modeling key anatomical landmarks and their spatial relationships, this approach reduces reliance on operator experience and may support safer and more efficient ultrasound-guided lumbar procedures. Future work will focus on expanding the dataset and validating the generalizability of the method across diverse clinical settings.

## 1 Introduction

In recent years, ultrasound-guidance has emerged as an increasingly valuable and promising tool in lumbar regional anesthesia and chronic pain management, including lumbar plexus block (1, 2), nerve root block (3, 4), medial branch block (5), facet joint injections (6, 7) and epidural steroid injections (8). Prior to the emergence of ultrasound-guidance, landmarked-based techniques were the primary approach in regional anesthesia (9), while radiographic visualization techniques, such as fluoroscopy and computed tomography (CT) were commonly used in chronic pain management (10, 11). Compared to these traditional guidance methods, ultrasonography offers several advantages like radiation-free, portable, and allows real-time visualization of the needle's trajectory. Moreover, it provides detailed visualization of anatomical structures, including bones, muscle layers, nerves, intrathecal structures, and blood vessels.

For lumbar spine injection procedures, two key transverse ultrasound views in the paravertebral region are widely used in clinical practice: the paramedian transverse view at the level of the transverse process (PTV-TP) (12, 13) and the paramedian transverse foraminal view (PTFV) (14, 15).

In the PTV-TP view, the transverse process is typically located at the center of the ultrasound image and appears as the stem of the shamrock, with the quadratus lumborum, psoas major, and erector spinae muscles forming the three surrounding “leaves” (16).

This view is fundamental to the Shamrock technique for lumbar plexus block (17), which has been reported to provide the best visualization of the lumbar plexus compared with other approaches (18). In addition, the PTV-TP view is also important in the quadratus lumborum block, as the clearly visualized transverse process serves as a reliable sonoanatomic landmark for accurate needle positioning (19).

The PTFV view, by contrast, is commonly used in the paramedian transverse scan (PMTS) technique for lumbar plexus block (20). This view helps to delineate the key anatomical structures relevant to the block, including the lumbar nerve roots, lumbar plexus, and the psoas compartment (21).

Moreover, visualization through the intervertebral foramen allows identification of intrathecal structures, such as the spinal canal, dura mater, and intrathecal space. Consequently, the PTFV provides a comprehensive ultrasound perspective including both paravertebral and intrathecal anatomy. Ultrasound-guided transforaminal injections based on the PTFV have been proposed as an effective treatment for lumbar radicular pain and provide an alternative access route to the spinal canal (22, 23).

Despite the advantages of ultrasound guidance, the complexity of lumbar sonoanatomy and the depth of target structures make these procedures technically challenging. Accurate identification of relevant anatomical landmarks is essential for procedural success and for minimizing complication. However, for novice anesthesiologists and pain physicians, reliable recognition of these ultrasound views requires substantial training and clinical experience. Therefore, automated detection and classification of PTV-TP and PTFV views from ultrasound images may provide valuable support for clinicians, particularly those with limited experience, and remains an unmet clinical need.

Recently, artificial intelligence (AI) has been extensively applied to medical ultrasound imaging for tasks such as image recognition and interpretation (24–26). However, automated recognition of lumbar ultrasound views remains challenging due to the complex spatial relationships among deep anatomical structures and the high visual similarity between adjacent scanning planes. Existing approaches often rely on end-to-end classification models that directly map ultrasound images to predefined view labels (27, 28). While such strategies can achieve reasonable performance under controlled conditions, they frequently lack interpretability and robustness when confronted with variations in probe position, patient anatomy, and image quality.

To address these challenges, we propose a two-stage framework that decouples anatomical localization from view classification. By first identifying shared anatomical landmarks through semantic segmentation and subsequently modeling their spatial configuration using coordinate-based features, the proposed framework enables robust and interpretable discrimination between PTV-TP and PTFV views.

## 2 Materials and methods

### 2.1 Dataset and annotation

In this study, a total of 425 patients scheduled for pain treatment at the Department of Anesthesiology, Peking Union Medical College Hospital, were involved. Written informed consent was obtained from each participant. The study was approved by the Institutional Review Board of Peking Union Medical College Hospital, Chinese Academy of Medical Sciences (Approval No. K1940).

Ultrasound scanning was performed prior to the initiation of pain treatment. All ultrasound images were acquired by an experienced anesthesiologist using a SonoSite X-Porte ultrasound system (Fujifilm, Tokyo, Japan) equipped with a curved-array low frequency transducer (2–5 MHz C60xp/5–2 Abdomen). The scanning depth was set to 7 cm and the gain to 50%. The resolution of the original images in this study was 960 × 720 pixels.

To obtain the PTV-TP image, patients were positioned in the lateral decubitus position. The ultrasound probe was placed on the flank just cranial to the iliac crest to identify the Shamrock sign, where the transverse process appears centrally on the ultrasound image. In this view, the spinous process and the vertebral body were simultaneously visualized in the same frame, showing a characteristic “thumb-up” appearance. Subsequently, the transducer was moved slightly cranially to the intervertebral level and directed medially to acquire the PTFV image. In this view, the ultrasound beam insonated the intervertebral foramen through the intertransverse space, clearly delineating the vertebral body, lamina, and spinous process, which together constitute the protrusion on the posterior surface of the vertebral body.

**Figure 1:**
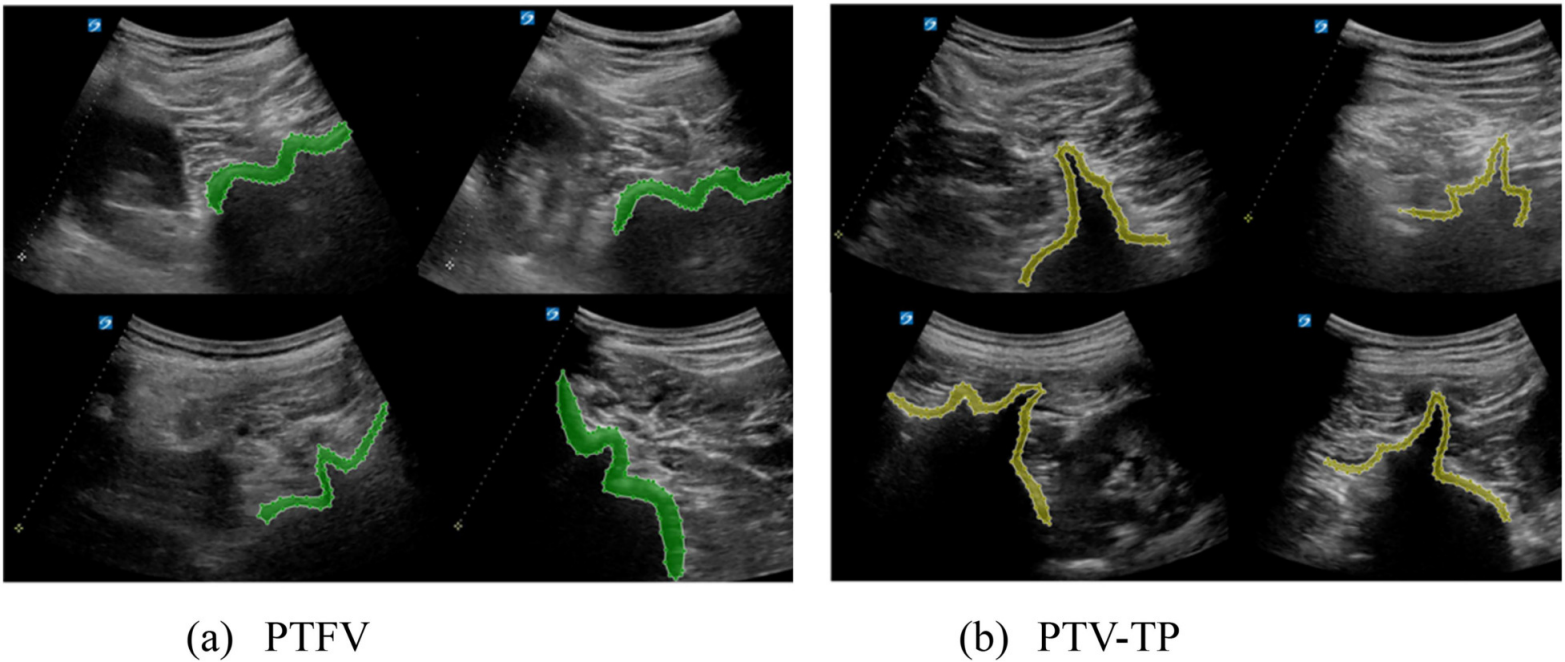
Illustration of the paramedian transverse foraminal view and transverse process view annotated by the professional anesthesiologist. **(a)** PTFV: the paramedian transverse foraminal view; **(b)** PTV-TP: the paramedian transverse view at the level of the transverse process.

All the images were independently reviewed by two anesthesiologists to confirm the target sonoanatomic structures. Images with insufficient quality due to fuzziness or incomplete anatomical representation were excluded from the dataset. Annotation was initially performed by one anesthesiologist who manually delineated the bone structures in both spine ultrasound views (PTV-TP and PTFV) by using an open-source annotation software LabelMe. The annotations were subsequently reviewed and verified by a senior anesthesiologist to ensure accuracy and inter-annotator consistency, as illustrated in Figure 1.

### 2.2 Model construction

Conventional instance segmentation models typically perform semantic segmentation and classification in a single stage, which requires a large amount of annotated data to achieve satisfactory performance. This requirement is particularly problematic in medical imaging, where datasets are often limited in size. These challenges are particularly pronounced in ultrasound imaging of the intervertebral foramen (denoted as F) and transverse process (denoted as T), due to blurred boundaries and minimal inter-class differences. Consequently, conventional semantic segmentation models often underperform on such datasets.

To address these limitations, we propose a novel two-stage segmentation framework for recognizing and distinguishing F and T in spine ultrasound images. In the first stage, F and T are treated as a single class, and a semantic segmentation model based on U-Net is trained. Subsequently, coordinate statistics are computed from the segmentation predictions of the training set to construct a new dataset containing spatial features derived from the segmentation results. In the second stage, a binary classifier is trained on this feature-based dataset to distinguish between F and T, thereby achieving the final instance segmentation.

Both stages of the proposed framework strictly followed the same patient-level data split, and the second-stage classifier was trained and evaluated only on segmentation results derived from the corresponding training, validation, and test sets to ensure data independence. Additionally, to benchmark the performance of our two-stage approach, we trained a standard CNN classifier directly on the classification task, serving as a baseline for comparison. This allows us to evaluate the effectiveness of the proposed two-stage approach relative to an end-to-end classification model.

1. First Stage: U-Net Semantic Segmentation

The U-Net architecture (29) is adopted in the first stage due to its strong performance in medical image segmentation. It consists of a symmetric encoder-decoder structure with skip connections, allowing for the integration of low-level spatial details with high-level semantic information. The encoder comprises convolutional and pooling layers, facilitating the extraction of feature representations from the image. Conversely, the decoder progressively restores the original resolution through up-sampling and convolution operations while amalgamating features from corresponding encoder levels, thereby yielding precise segmentation outcomes.

**Figure 2:**
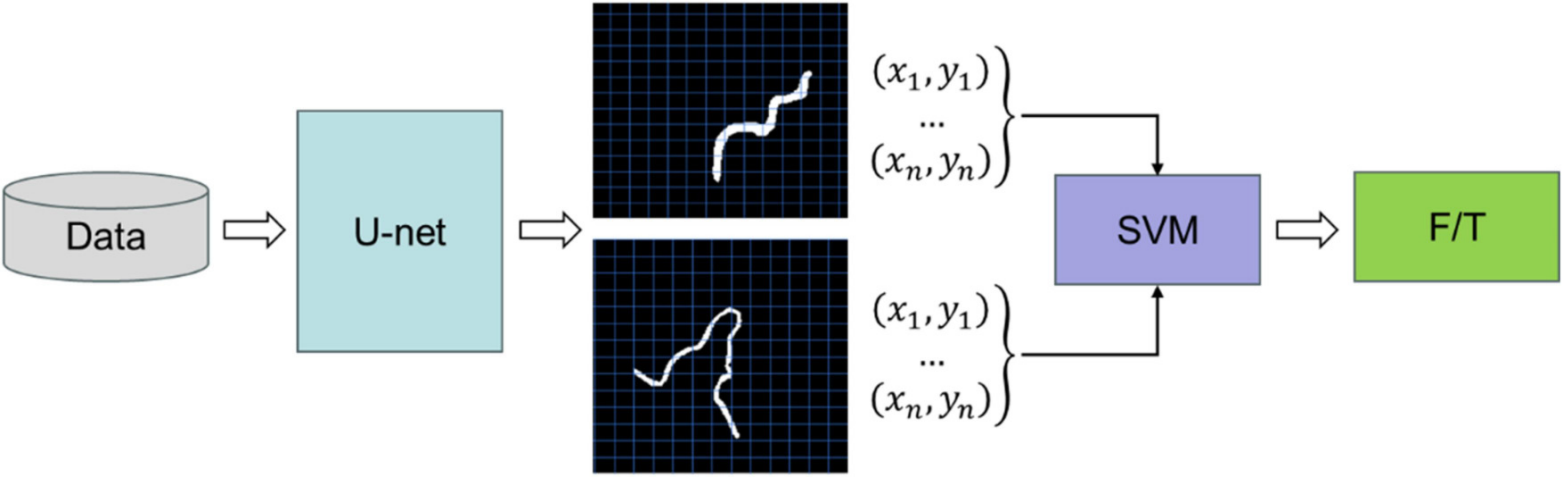
Schematic diagram of nerve root segmentation and classification; F: intervertebral foramen; T: transverse process.

In this stage, F and T were treated as a single category (as illustrated in Figure 2). The advantage of this approach allows the model to focus on distinguishing target from the background. This simplification reduces the annotation complexity and improves segmentation performance, especially in scenarios with limited training data. In comparison to conventional instance segmentation models, the segmentation output from this stage provides accurate masks of the target regions, which are then used for classification in the second stage.

2. Second Stage: Coordinate-Based SVM Classifier

Based on clinical knowledge and observation, F and T present distinct geometric characteristics. The typical shape of F resembles a “goat peak” with two peaks and one trough, displaying a smooth and regular structure with few inflection points. In contrast, T generally appears more irregular, with a distorted shape and multiple inflection points. These shape-based features can be effectively used to differentiate F from T.

**Figure 3:**
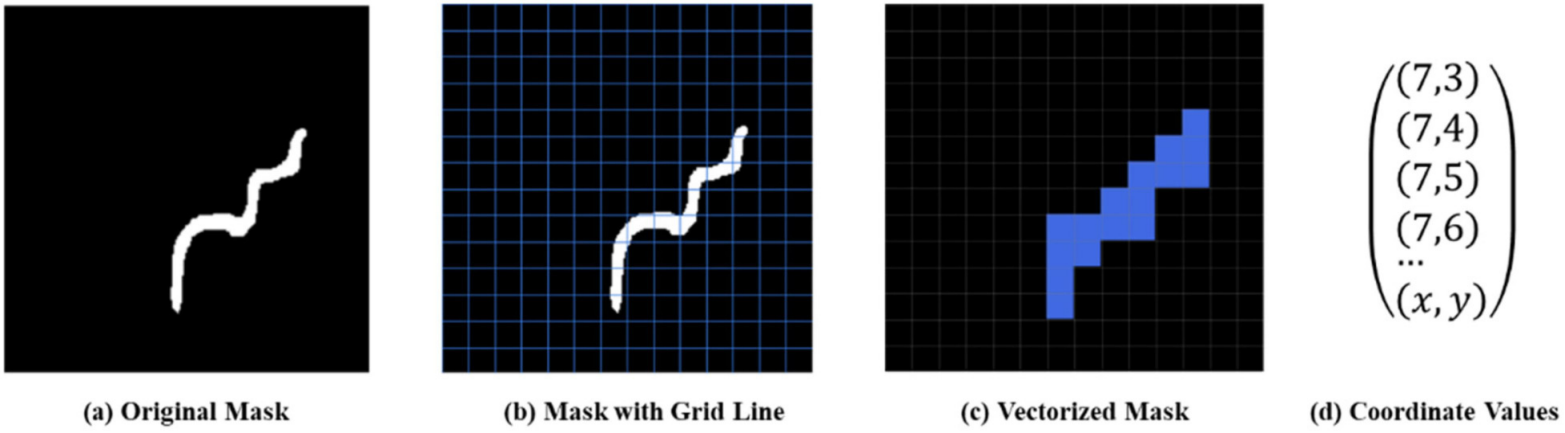
Process of constructing a coordinate-based feature dataset from semantic segmentation results. **(a)** Original mask, **(b)** mask with grid line, **(c)** vectorized mask, and **(d)** coordinate values.

A segmentation mask is a binary image with the same size as the input, where pixel values indicate class membership. Since F and T are treated as a single class in the first stage, the segmentation mask contains only two-pixel values (0 and 1). To create features for the SVM classifier, each 224 × 224 segmentation mask was divided into 16 × 16 non-overlapping subregions, resulting in 256 subregions per image, as shown in Figure 3. A binary feature vector $F_{i}=[c_{0},c_{1},\ldots ,c_{255}]$ was then defined, where $c_{j}=1$ if subregion *j* contains at least one pixel labeled as 1, and $c_{j}=0$ otherwise. This transformation yields a feature matrix of size N×L, where N=425 is the total size of our collected dataset and L=256 is the feature dimension.

Given that the coordinate-based features exhibit class-dependent patterns across the feature space, a Support Vector Machine (SVM) classifier was employed in the second stage. The SVM constructs hyperplanes to separate the two categories, F and T, based on their spatial characteristics. The classifier then assigns labels to the segmented regions from the first stage, thus completing the instance segmentation task.

### 2.3 Dataset splitting and evaluation metric

The dataset was divided at the patient level by using patient identifiers to ensure that all ultrasound images from a given individual were assigned exclusively to one of the training, validation, or testing subsets (70%, 10%, and 20%, respectively). This strategy was adopted to prevent data leakage caused by correlated samples from the same subject and to ensure independence across subsets. The same split was consistently applied to both the U-Net segmentation model in the first stage and the SVM classifier in the second stage, enabling a fair evaluation of the entire two-stage framework.

For the segmentation task, Intersection over Union (IoU) metric was used to evaluate the performance of the U-Net model. IoU is a widely used indicator in image segmentation, measuring the degree of overlap between the predicted segmentation and the ground truth. It quantifies the similarity between the two regions by calculating the ratio of their intersection over their union. The IoU is calculated as follows:

$$\mathrm{IoU}=\frac{\mathrm{Area}\;\mathrm{of}\;\mathrm{Intersection}}{\mathrm{Area}\;\mathrm{of}\;\mathrm{Union}}\;\;\;\;\;\;\;\;\;\;\;\;\;\;\;\;\;\;\;\;\;\;(1)$$

where the intersection refers to the overlapping area between the predicted region and the ground truth, and the union refers to the total area covered by both. For the classification task, accuracy was employed as the evaluation metric. Accuracy is one of the most commonly used metrics in classification problems. It represents the proportion of correctly classified samples to the total number of samples.

## 3 Results

After training the classifier on the newly constructed coordinate-based dataset, promising performance was achieved in classification tasks. To evaluate the effectiveness of the proposed grid-based SVM classifier in the second stage, its performance was compared with a standard CNN classifier trained directly for the classification task. Table 1 summarizes the segmentation IoU and classification accuracy of the different models.

**Table 1:** Segmentation and classification performance of different models.

Model	Segmentation IoU (%)	Classification accuracy (%)
U-Net (SSeg)+ SVM	83.21	97.66
U-Net (SSeg)	83.21	95.34
U-Net (MCSeg)	77.33	82.75
CNN (Baseline)	/	85%

IoU, intersection over union; SVM, support vector machine; SSeg, semantic segmentation; MCSeg, multi-class semantic segmentation; CNN, convolutional neural network.

As shown in Table 1, the CNN classifier achieved a classification accuracy of 85%, whereas the proposed two-stage framework achieved an accuracy of 97.66%, indicating the superior performance of this method in distinguishing F and T under limited-sample conditions. In terms of segmentation performance, the U-Net multi-class semantic segmentation model, which treated F and T as separate categories, achieved an IoU of 77.33%. In contrast, when F and T were merged into a single category during semantic segmentation, the U-Net model achieved a substantially higher IoU of 83.21%. These results suggest that treating F and T as one category in the first-stage segmentation task helps the model to learn more robust target–background representations, thereby improving segmentation quality. The improved segmentation results subsequently contribute to more accurate classification in the second stage.

**Figure 4:**
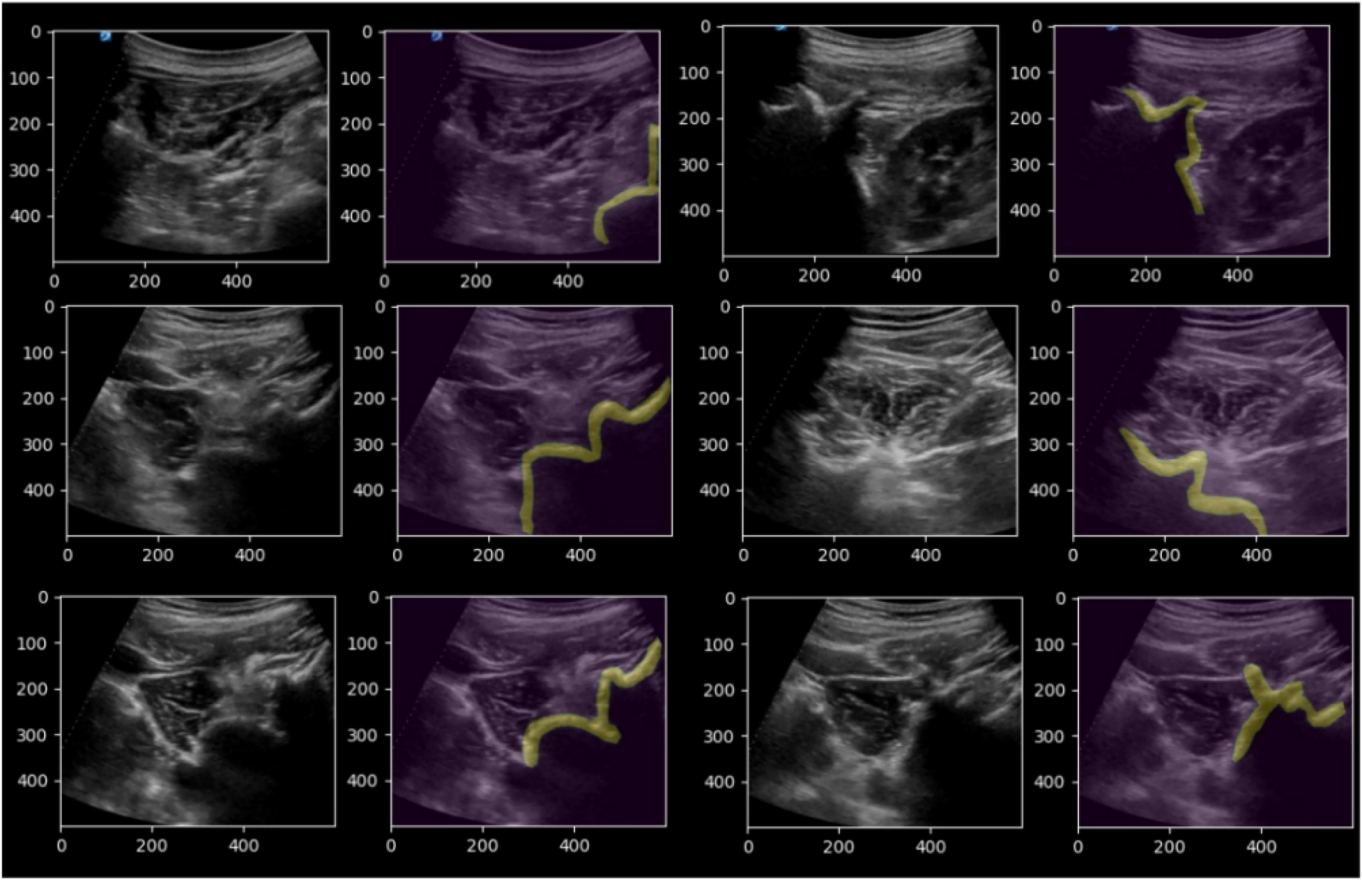
Visualization of U-net semantic segmentation results.

In addition, we provide representative visualization examples in Figure 4, illustrating the performance of the U-net Semantic Segmentation model across various scenarios. These examples highlight the model's ability to accurately identify and segment target anatomical structures, even under challenging imaging conditions.

Overall, the experimental results demonstrate that the proposed two-stage framework: combining semantic segmentation with coordinate-based classification, can effectively segment and classify lumbar spine ultrasound structures. The observed improvements in both segmentation accuracy and classification performance support the feasibility of this approach for automated lumbar ultrasound view recognition.

## 4 Discussion

In this study, we proposed a two-stage deep learning framework for automated segmentation and classification of two clinically important mid-lumbar transverse ultrasound views, namely PTV-TP and PTFV. The experimental results demonstrate that the proposed approach can accurately delineate key lumbar bony structures and reliably distinguish between these two views. Accurate identification of PTV-TP and PTFV is clinically critical, as the PTV-TP view provides essential anatomical landmarks for lumbar plexus and quadratus lumborum blocks, while the PTFV view enables visualization of the intervertebral foramen, nerve roots, and related intrathecal structures. Given that more than ten distinct ultrasound views may be encountered in lumbar scanning (30–32), recognizing these specific views remains particularly challenging for novice anesthesiologists and pain physicians. By automating this recognition process, the proposed framework has the potential to reduce the learning curve, minimize identification errors, and promote standardization of ultrasound-guided lumbar procedures in routine clinical practice.

Compared with conventional end-to-end classification approaches, the proposed two-stage framework demonstrated clear advantages in robustness and interpretability. Instead of directly mapping ultrasound images to predefined view labels, our method explicitly separates anatomical localization from view classification. This design aligns with clinical reasoning, in which stable bony landmarks are first identified and then used to infer the corresponding scanning plane.

In lumbar ultrasound imaging, image quality is frequently degraded by speckle noise, acoustic shadowing, and blurred tissue boundaries, particularly in deep anatomical regions. End-to-end classification models tend to rely heavily on global texture patterns, making them vulnerable to such artifacts and to variations in probe positioning and patient anatomy. In contrast, by focusing on bone-based structures and their spatial configuration, our approach reduces dependence on complex soft-tissue appearances and improves tolerance to image quality variability.

Moreover, the coordinate-based classification strategy improves model transparency by allowing the classification decision to be traced back to spatial distributions of segmented anatomical structures. This characteristic is particularly valuable for clinical assistance and medical education, as it enables users to understand not only the predicted view label but also the anatomical rationale underlying the prediction.

An additional advantage of the proposed framework lies in its ability to operate effectively under limited data conditions. In the first stage, treating the intervertebral foramen (F) and transverse process (T) as a single category simplifies the segmentation task to a target-versus-background problem. This strategy reduces annotation complexity and allows the U-Net model to learn more robust representations of lumbar bony structures, resulting in improved segmentation performance compared with conventional multi-class semantic segmentation. The improved segmentation quality provides a reliable foundation for subsequent classification and highlights the importance of task decomposition in medical image analysis, particularly when annotated datasets are small.

In the second stage, coordinate-based spatial features derived from the segmentation masks were used for classification. Clinically, F and T exhibit distinct geometric and spatial characteristics, which are not always captured effectively by purely appearance-based models. By encoding the spatial distribution of segmented regions into a grid-based feature representation, the proposed method enables effective discrimination between PTV-TP and PTFV views. The SVM classifier demonstrated strong performance in this setting, outperforming a baseline CNN classifier trained directly on the classification task. This result suggests that, for small-sample ultrasound datasets, combining deep learning-based feature extraction with classical machine learning classifiers can offer a favorable balance between accuracy, robustness, and computational efficiency. Furthermore, the coordinate-based classification strategy improves interpretability, as classification decisions can be traced back to anatomically meaningful spatial patterns, which is valuable for both clinical assistance and medical education.

From a practical perspective, the proposed framework exhibits good scalability and potential for clinical deployment. Once trained, the model can be integrated into real-time ultrasound systems to provide on-screen guidance for view recognition, assisting clinicians during scanning and needle placement. By standardizing view identification, the framework may contribute to improved procedural safety and consistency, particularly in training environments and settings with limited expert availability.

Despite these promising results, several limitations should be acknowledged. First, the dataset size was relatively limited and may not fully represent the diversity of patient populations, including those of different age groups and body types. Second, all ultrasound images were acquired at a single center by a single experienced physician using the same high-end ultrasound system. In real-world clinical settings, image quality and appearance can vary substantially due to differences in equipment, operators, and patient-related factors, which may limit the generalizability of the current model. Future studies should therefore incorporate multi-center, multi-operator datasets to further validate robustness and clinical applicability. Finally, although the proposed framework demonstrated resilience to common ultrasound artifacts, its performance may still be affected by severely degraded image quality. Future work may explore more advanced algorithms to further improve robustness under challenging imaging conditions.

## 5 Conclusion

In this study, we proposed a two-stage framework for automated segmentation and classification of the target lumbar bony structures to assist in the recognition of two clinically important mid-lumbar transverse ultrasound views, namely PTV-TP and PTFV. By decoupling anatomical localization from view classification, the proposed approach effectively addresses the challenges posed by complex lumbar sonoanatomy and limited annotated data.

In the first stage, treating the intervertebral foramen and transverse process as a unified category enabled robust semantic segmentation, achieving an IoU of 83.21%. In the second stage, coordinate-based spatial features derived from the segmentation masks allowed accurate discrimination between F and T, resulting in a classification accuracy of 97.66%. These results demonstrate that combining deep learning-based segmentation with spatially interpretable classification can provide reliable and transparent performance for lumbar ultrasound view recognition.

The proposed framework has the potential to help novice clinicians by reducing the learning curve, improving consistency in view identification, and improving the safety and efficiency of ultrasound-guided lumbar procedures. Future work should focus on incorporating more diverse, multi-center datasets and validating the framework across different clinical settings to further improve the robustness and generalizability of algorithm.

## Data Availability

The datasets presented in this article are not readily available because this dataset is not allowed shared without the permission of hospital. Requests to access the datasets should be directed to cuixulei10685@pumch.cn.

## References

[1] Lifei L Shangyingying L Yanzhe T. The application of ultrasound guidance in clinical teaching of lumbar Plexus block in children. China Cont Med Educ. (2018) 10(2):34–6. 10.3969/j.issn.1674-9308.2018.02.017

[2] Xiao J Fang Y Yu Y Li J Luo Y-r Liu Y Ultrasound guidance and nerve stimulation combined versus nerve stimulation alone for lumbar Plexus block: a randomized controlled trial. Curr Med Sci. (2020) 40(6):1182–90. 10.1007/s11596-020-2307-9 33428148

[3] Chumnanvej S Kittayapirom K Chumnanvej S. Visualization of needle-tip localization by ultrasound guidance with contrast bubble in lumbar selective nerve root block: clinical pilot study. World Neurosurg. (2018) 111(March):e418–23. 10.1016/j.wneu.2017.12.079 29274452

[4] Di W Yanshi H Dawei S. Effect of cervical nerve root block on cervical spondylosis under ultrasound guidance. J Clin Psychosomatic Dis. (2018) 24(4):154–56. 10.3969/j.issn.1672-187X.2018.04.047

[5] Nisolle M-L Ghoundiwal D Engelman E El Founas W Gouwy J Guntz E Comparison of the effectiveness of ultrasound guided versus fluoroscopy-guided medial lumbar bundle branch block on pain related to lumbar facet joints: a multicenter randomized controlled non-inferiority study. BMC Anesthesiol. (2023) 23(1):1–8. 10.1186/s12871-023-02029-9 36906521 PMC10007783

[6] Tay M Sim Hwei Sian SC Eow CZ Ho KLK Ong JH Sirisena D. Ultrasound- guided lumbar spine injection for axial and radicular pain: a single institution early experience. Asian Spine J. (2021) 15(2):216–23. 10.31616/asj.2019.0399 32872762 PMC8055452

[7] Ye L Wen C Liu H. Ultrasound-Guided ver- sus low dose computed tomography scanning guidance for lumbar facet joint injections: same accuracy and efficiency. BMC Anesthesiol. (2018) 18(1):1–7. 10.1186/s12871-018-0620-7 30404599 PMC6223004

[8] Kumar Hazra A Bhattacharya D Mukherjee S Ghosh S Mitra M Mandal M. Ultra-sound versus fluoroscopy-guided caudal epidural steroid injection for the treatment of chronic low back pain with radiculopathy: a ran- domised, controlled clinical trial. Indian J Anaesth. (2016) 60(6):388–92. 10.4103/0019-5049.183391 27330199 PMC4910477

[9] Jeyakumar A Weaver JJ Chick JFB Hage AN Koo KSH Shivaram GM Spinal ultrasound after failed landmarked-based lumbar puncture: a single institutional experience. Pediatr Radiol. (2021) 51(2):289–95. 10.1007/s00247-020-04831-w 32940728

[10] Koizuka S Nakajima K Mieda R. CT-Guided Nerve block: a review of the features of CT fluoroscopic guidance for nerve blocks. J Anesth. (2014) 28(1):94–101. 10.1007/s00540-013-1675-8 23873005

[11] Wang D. Image guidance technologies for interventional pain procedures: ultrasound, fluoroscopy, and CT. Curr Pain Headache Rep. (2018) 22(1):0. 10.1007/s11916-018-0660-1 29374352

[12] Kim YW Yoo S Park SK Bae H Lim YJ Kim JT. Optimal angle of needle insertion for spinal anesthesia in patients with spondylolisthesis: an ultrasonographic study. BMC Anesthesiol. (2021) 21(1):221. 10.1186/s12871-021-01444-0 34496754 PMC8424909

[13] Duniec L Nowakowski P Kosson D Łazowski T. Anatomical landmarks based assessment of intravertebral space level for lumbar puncture is misleading in more than 30%. Anaesthesiol Intensive Ther. (2013) 45(1):1–6. 10.5603/AIT.2013.0001 23572300

[14] Yeo CG Jeon I Kim SW Ko SK Woo BK Song KC. Three-Years outcome of microdiscectomy via paramedian approach for lumbar foraminal or extraforaminal disc herniations in elderly patients over 65 years old. Korean J Spine. (2016) 13(3):107–13. 10.14245/kjs.2016.13.3.107 27799988 PMC5086460

[15] Bae JS Kang KH Park JH Lim JH Jang IT. Postoperative clinical outcome and risk factors for poor outcome of foraminal and extraforaminal lumbar disc herniation. J Korean Neurosurg Soc. (2016) 59(2):143–48. 10.3340/jkns.2016.59.2.143 26962420 PMC4783480

[16] Nielsen MV Bendtsen TF Børglum J. Superiority of ultrasound-guided shamrock lumbar plexus block. Minerva Anestesiol. (2018) 84(1):115–21. 10.23736/S0375-9393.17.11783-9 28749094

[17] Sauter AR. The “Shamrock Method”-a new and promising technique for ultrasound guided lumbar plexus blocks. Br J Anaesth. (2013) 111(eLetters):el9814. 10.1093/bja/el_9814

[18] Pangthipampai P Tangwiwat S Pakpirom J Songthamwat B Karmakar MK. Ultrasound visualization of the anatomy relevant for lumbar plexus block: comparison of the paramedian transverse and shamrock scan technique. Reg Anesth Pain Med. (2019) 44(6):634–8. 10.1136/rapm-2018-100011 30886068

[19] Balocco AL López AM Kesteloot C Horn JL Brichant JF Vandepitte C Quadratus lumborum block: an imaging study of three approaches. Reg Anesth Pain Med. (2021) 46(1):35–40. 10.1136/rapm-2020-101554 33159007

[20] Karmakar MK Li JW Kwok WH Hadzic A. Ultrasound-guided lumbar plexus block using a transverse scan through the lumbar intertransverse space: a prospective case series. RegAnesth Pain Med. (2015) 40(1):75–81. 10.1097/AAP.0000000000000168 25469756

[21] Karmakar MK Li JW Kwok WH Soh E Hadzic A. Sonoanatomy relevant for lumbar plexus block in volunteers correlated with cross-sectional anatomic and magnetic resonance images. Reg Anesth Pain Med. (2013) 38(5):391–7. 10.1097/AAP.0b013e31829e52cc 23974865

[22] Khalaf AM Yedavalli V Massoud TF. Magnetic resonance imaging anatomy and morphometry of lumbar intervertebral foramina to guide safe transforaminalsubarachnoid punctures. Clin Anat. (2020) 33(3):405–13. 10.1002/ca.23533 31837183

[23] Bhatia A Flamer D Shah PS Cohen SP. Transforaminalepidural steroid injections for TreatingLumbosacral radicular pain from herniated inter- vertebral discs: a systematic review and meta-analysis. Anesth Analg. (2016) 122(3):857–70. 10.1213/ANE.0000000000001155 26891397

[24] Chin KJ Karmakar MK Peng P. Ultrasonography of the adult thoracic and lumbar spine for central neuraxial blockade. Anesthesiology. (2011) 114(6):1459–85. 10.1097/ALN.0b013e318210f9f8 21422997

[25] Chi M Chen AS. Ultrasound for lumbar spinal procedures. Phys Med Rehabil Clin N Am. (2018) 29(1):49–60. 10.1016/j.pmr.2017.08.005 29173664

[26] Zeng Y Tsui PH Pang K Bin G Li J Lv K MAEF-Net: multi-attention efficient feature fusion network for left ventricular segmentation and quantitative analysis in two-dimensional echocardiography. Ultrasonics. (2023) 127:106855. 10.1016/j.ultras.2022.106855 36206610

[27] Huang C Zhou Y Tan W Qiu Z Zhou H Song Y Applying deep learning in recognizing the femoral nerve block region on ultrasound images. Ann Transl Med. (2019) 7(18):453. 10.21037/atm.2019.08.61 31700889 PMC6803209

[28] Xing W He C Li J Qin W Yang M Li G Automated lung ultrasound scoring for evaluation of coronavirus disease 2019 pneumonia using two-stage cascaded deep learning model. Biomed Signal Process Control. (2022) 75:103561. 10.1016/j.bspc.2022.103561 35154355 PMC8818345

[29] Shin Y Yang J Lee YH Kim S. Artificial intelligence in musculoskeletal ultrasound imaging. Ultrasonography. (2021) 40(1):30–44. 10.14366/usg.20080 33242932 PMC7758096

[30] Hetherington J Lessoway V Gunka V Abolmaesumi P Rohling R. SLIDE: automatic spine level identification system using a deep convolutional neural network. IntJ Comput Assist Radiol Surg. (2017) 12(7):1189–98. 10.1007/s11548-017-1575-8 28361323

[31] Pesteie M Lessoway V Abolmaesumi P Rohling R. Automatic midline identification in transverse 2-D ultrasound images of the spine. Ultrasound Med Biol. (2020) 46(10):2846–54. 10.1016/j.ultrasmedbio.2020.04.018 32646685

[32] Ronneberger O Fischer P Brox T. U-net: convolutional networks for biomedical image segmentation. InMedical Image Computing and Computer-Assisted Intervention–MICCAI 2015: 18th International Conference, Munich, Germany, October 5-9, 2015, Proceedings, Part III 18. Springer (2015). p. 234–2

